# Recurrent Hyperkalemia During General Anesthesia in a Dog

**DOI:** 10.3389/fvets.2020.00210

**Published:** 2020-04-24

**Authors:** Carissa W. Tong, Anusha Balakrishnan, Rachel Matusow Wynne

**Affiliations:** ^1^Department of Emergency and Critical Care, Cornell University Veterinary Specialists, Stamford, CT, United States; ^2^Department of Ophthalmology, Cornell University Veterinary Specialists, Stamford, CT, United States

**Keywords:** propofol, hyperkalemia, propofol infusion syndrome, anesthesia, canine

## Abstract

**Objective:** To describe the development of recurrent hyperkalemia in a dog that underwent general anesthesia at two different hospitals within a month. The definitive underlying cause of the hyperkalemia remains unknown.

**Case summary:** A 11 year-old male neutered Rottweiler underwent general anesthesia on two separate occasions at two different hospitals for ophthalmic surgery within a month and developed marked hyperkalemia on each occasion. The patient received similar drug protocols in both instances, including propofol, midazolam, non-depolarizing neuromuscular blocking agents, and isoflurane inhalant anesthetic. The patient showed ECG changes consistent with hyperkalemia during the first anesthetic event, but not the second. No underlying cause of hyperkalemia was definitively identified. The patient responded to standard therapy for hyperkalemia on both occasions and serum potassium levels returned to normal. The patient was discharged from the hospital without further complications and post-operative rechecks showed persistently normal serum potassium levels.

**New or unique information provided:** Considering that there is a relationship between the development of severe hyperkalemia and propofol administration in human patients, it is possible that such a relationship exists in veterinary patients. However, numerous other diseases and medications can also lead to peri-operative hyperkalemia. Veterinary professionals should be aware that hyperkalemia can develop intra-operatively and remains be an important differential diagnosis in bradycardic patients under anesthesia that are not responding to traditional therapies.

## Introduction

Hyperkalemia is a potentially life-threatening electrolyte abnormality defined as a serum potassium level >5.5 mmol/L in dogs (1). It is an uncommon intra-operative complication but can occur secondary to a variety of causes. Recently, there have been increased reports of the development of hyperkalemia in canine patients under anesthesia (2–4). This case report describes the development of repeated severe hyperkalemia under general anesthesia in a dog of which the cause remains unknown.

## Case Presentation

An 11-year-old, 49.9 kg, male neutered Rottweiler was presented to the Ophthalmology Service of Cornell University Veterinary Specialists (CUVS) for an elective bilateral phacoemulsification of diabetes mellitus-induced cataracts. The patient had an extensive medical history of the following: historical facial trauma resulting in right-sided facial nerve paralysis and right-sided lagophthalmos, inflammatory bowel disease, diabetes mellitus with episodes of diabetic ketoacidosis, bilateral cranial cruciate ligament rupture with bilateral tibial plateau leveling osteotomies, recurrent urinary tract infections, an acute intervertebral disc extrusion necessitating a C6–C7 ventral slot surgery, and a splenectomy for a splenic mass with histopathology confirming extramedullary hematopoiesis. He had a history of mild, intermittent hyperkalemia (5.6–5.9 mmol/L) that was suspected to be pseudohyperkalemia secondary to thrombocytosis. Other causes of this mild, intermittent hyperkalemia had previously been ruled out through extensive diagnostic testing, including an abdominal ultrasound, thoracic and abdominal computerized tomography, as well as endocrine testing (resting cortisol levels, adrenocorticotropic hormone [ACTH] stimulation tests, and serum lead levels). Pre-operative blood work performed 3 weeks prior to his cataract surgery with his primary care veterinarian was submitted to an external laboratory^1^ and revealed mild hypoalbuminemia, elevated alkaline phosphatase, hyperglycemia, hyponatremia, mild hyperkalemia, and hypercholesterolemia (Table 1). His platelet count was normal (Table 1). His fructosamine level was normal (333 umol/L; reference interval 136–350 umol/L) which indicated good diabetic regulation (<360 umol/L). Despite the adequate fructosamine level, a 24-h blood glucose curve performed 2 days after his pre-operative blood work showed a blood glucose range of 21–36 mmol/L. As such, his Neutral Protamine Hagedorn (NPH) insulin dose was increased to 30 units subcutaneously (SQ) every 12 h.

**Table 1:** Pre-operative bloodwork at primary care veterinarian 3 weeks prior to anesthesia.

Albumin (g/dL) (RI 27–44 g/dL)	25
ALT (U/L) (RI 12–118 U/L)	46
ALP (U/L) (RI 5–131 U/L)	148
Glucose (mmol/L) (RI 3.8–7.7 mmol/L)	32.9
Sodium (mmol/L) (RI 139–154 mmol/L)	137
Potassium (mmol/L) (RI 3.6–5.5 mmol/L)	5.6
Cholesterol (mmol/L) (RI 2.39–8.42 mmol/L)	18.4
Amylase (U/L) (RI 290–1125 U/L)	441
Phosphorus (mmol/L) (RI 0.8–1.93 mmol/L)	1.35
Calcium (mmol/L) (RI 2.22–2.84 mmol/L)	2.27
Hematocrit (%) (RI 36–60%)	38
Platelet count (10^3^/uL) (RI 170–400 10^3^/uL)	292

*RI, reference interval; ALP, alkaline phosphatase; ALT, alanine transaminase*.

On admission to the hospital, his heart rate (HR) (136 beats/min; reference interval, 80 to 140 beats/min) and body temperature (38.1°C; reference interval, 37.8° to 39.5°C) were within normal limits, but his respiratory rate was elevated as he was panting. This patient was aggressive and always had a higher resting HR documented during his previous 41 visits to the hospital (128–168 bpm) which was attributed to anxiousness. Physical examination revealed normal cardiothoracic auscultation. A complete ophthalmic examination was performed by a board-certified veterinary ophthalmologist including slit lamp biomicroscopy, rebound tonometry, fluorescein staining, electroretinography, and posterior segment ocular ultrasound. He had received NPH insulin^2^ SQ (15 IU) at half of his normal dose 3 h prior to admission. He had received ketorolac^3^ 1 guttae (ggt) in both eyes (OU), prednisolone acetate^4^ 1 ggt OU, and Genteal^5^ $\frac{1}{4}$ strip OU 12 h prior to admission. His blood glucose at presentation was 21 mmol/L (reference interval 4–9.7 mmol/L). Pre-operatively, per standard ophthalmology protocol, he received a total of four doses each of ketorolac, prednisolone acetate, tropicamide^6^, and neomycin-polymyxin B-gramicidin^7^ OU. He also received one dose each of phenylephrine^8^ and dorzolamide-timolol^9^ OU.

A peripheral intravenous catheter^10^ was placed and the patient was premedicated with methadone^11^ (0.3 mg/kg) and midazolam^12^ (0.2 mg/kg) administered intravenously (IV). General anesthesia was induced with propofol^13^ (1 mg/kg) IV to allow endotracheal intubation. The patient was connected to a rebreathing anesthetic circuit with isoflurane^14^ as the inhalant anesthetic and was mechanically ventilated throughout the procedure. Cefazolin^15^ was given at the beginning of surgery as a peri-operative prophylactic antimicrobial agent. Monitoring consisted of continuous electrocardiography (ECG), esophageal thermometry, capnography, pulse oximetry, and indirect blood pressure measurement^16^. Once the dog was anesthetized and positioned in dorsal recumbency for surgery, cisatracurium besylate^17^ (0.2 mg/kg each time) was given IV 35- and 50-min following induction of anesthesia. Train-of-four monitoring was performed in the superficial peroneal nerve to assess depth of neuromuscular blockade. Fluid therapy was provided throughout the procedure with Plasmalyte^18^ (10 mL/kg/hr). The patient's HR remained between 100 and 120 bpm initially and his systolic blood pressure (SBP) between 100 and 120 mmHg as measured by an indirect oscillometric device^16^. Phacoemulsification and intraocular lens implantation of the left eye were completed uneventfully. Patient positioning was adjusted slightly after completion of the surgical procedure on the left eye to bring the right eye into appropriate position under the surgical microscope. Ninety minutes following propofol induction and 55 min following cisatracurium administration, a marked, acute decrease in his HR (25–30 bpm) and blood pressure (SBP 65–70 mmHg) were noted during phacoemulsification of the right lens. His end-tidal carbon dioxide was 39 mmHg. Given the onset of his bradycardia and hypotension, and the nature of surgery, iatrogenic triggering of the oculo-cardiac reflex was considered a possibility; therefore, anticholinergic medications, both glycopyrrolate^19^ (0.02 mg/kg) and subsequently atropine^20^ (0.02 mg/kg), were given IV. However, as the patient's eye was not being actively manipulated and no improvement was noted with anticholinergic medications, oculo-cardiac reflex was considered less likely. The bradycardia and hypotension did not respond to administration of a crystalloid fluid bolus (10 mL/kg Plasmalyte), decreasing inhalant anesthetic concentration, or antagonism of his previously administered methadone and midazolam with intravenous administration of naloxone^21^ (0.02 mg/kg) and flumazenil^22^ (0.01 mg/kg), respectively. A venous blood gas sample^23^ obtained from his cephalic vein revealed metabolic acidosis with marked hyperkalemia and relative hyponatremia (Table 2). His blood glucose and lactate were normal (Table 2). Throughout this period, his ECG demonstrated sinus bradycardia with absent P waves and tented T waves, consistent with changes secondary to hyperkalemia. The patient received 10% calcium gluconate^24^ (0.15 mL/kg) IV for cardioprotection. His bradycardia rapidly resolved following administration of calcium gluconate. Regular insulin^25^ (0.12 IU/kg) and terbutaline^26^ (0.01 mg/kg) were also subsequently administered IV to facilitate potassium transport into the intracellular fluid compartment. As the bradycardia resolved, so did the hypotension (SBP 110–120 mmHg). Recheck venous blood gas analysis an hour later revealed mild improvement in his metabolic acidosis, hyperkalemia, and hyponatremia (Table 2). An in-house chemistry panel^27^ was checked and revealed moderate hyperglycemia, mild hyperphosphatemia, mild hypoalbuminemia, hypercholesterolemia and a mildly low amylase (Table 3).

**Table 2:** Point of care bloodwork (venous) at CUVS.

**Date/Time**	**12/06/2018** **12:40** **During isoflurane anesthesia when bradycardia occurred**	**12/06/2018** **13:30** **1-h following anesthesia**	**12/06/2018** **21:20** **9-h following anesthesia**	**12/07/2018** **08:30** **20-h following anesthesia**	**12/10/2018** **4 days following anesthesia**
Venous pH (RI 7.32–7.44)	7.178	7.250	7.375	7.435	–
pCO_2_ (mmHg) (RI 39–47 mmHg)	40.2	37	30.8	28.3	–
${\text{HCO}}_{3}^{-}$ (mmol/L) (RI 18–26 mmol/L)	14.8	15.9	17.6	18.6	–
BE (mmol/L) (RI −5 to +1 mmol/L)	−13.1	−10.5	−6.4	−4.4	–
Glucose (mmol/L) (RI 3.5–6.16 mmol/L)	16.7	18.1	15	9.8	23.4
Sodium (mmol/L) (RI 140–150 mmol/L)	134.3	138.8	143	143.3	145.1
Potassium (mmol/L) (RI 3.9–4.9 mmol/L)	8.08	6.08	4.08	4.4	4.6
Chloride (mmol/L) (RI 109–120 mmol/L)	112	114	111	113	104
iCa (mmol/L) (RI 1.2–1.5 mmol/L)	1.21	1.27	1.19	1.18	1.22
Lactate (mmol/L) (RI 0.5–2 mmol/L)	1.58	1.57	1.55	1.67	2.3

*RI, reference interval; pCO_2_, partial pressure of carbon dioxide; ${\text{HCO}}_{3}^{-}$, bicarbonate; BE, base excess; iCa, ionized calcium*.

**Table 3:** In-house chemistry panel at CUVS 1-h following anesthesia.

Albumin (g/dL) (RI 27–39 g/dL)	23
ALT (U/L) (RI 18–121 U/L)	38
ALP (U/L) (RI 5–160 U/L)	102
Glucose (mmol/L) (RI 3.5–6.3 mmol/L)	15
Cholesterol (mmol/L) (RI 3.4–8.97 mmol/L)	14.6
Amylase (U/L) (RI 337–1469 IU/L)	835
Phosphorus (mmol/L) (RI 0.8–1.9 mmol/L)	1.55
Calcium (mmol/L) (RI 2.09–2.94 mmol/L)	2.14

*RI, reference interval; ALP, alkaline phosphatase; ALT, alanine transaminase*.

In order to further investigate the cause of his acute hyperkalemia, additional diagnostic testing performed included an abdominal ultrasound which revealed static hyperechoic hepatomegaly with heterogeneous hepatic echotexture, mild bilateral adrenomegaly, a small left renal cortical cyst, and an absent spleen. An ACTH stimulation test^28^ was performed and revealed no evidence of hypoadrenocorticism or hyperadrenocorticism (pre-cortisol: 132.5 nmol/L, post-cortisol: 182.2 nmol/L). A recheck venous blood gas obtained 9 h later revealed resolution of the metabolic acidosis, hyperkalemia and hyponatremia (Table 2). The patient recovered well from anesthesia and his surgical procedure. He was discharged from the hospital 26 h post-operatively and remained stable at home. Recheck electrolytes measured 4 days post-operatively were within normal limits (Table 2).

Over the next 2 weeks, the patient was re-evaluated several times and remained normokalemic. However, his chemistry panel^28^ showed that he remained mildly hypoalbuminemic and hypercholesterolemic (Table 4). His phosphorus and amylase levels normalized (Table 4). Moderate intraocular pressure elevation was detected 1 week after surgery, and dorzolamide-timolol ophthalmic solution was prescribed to be applied to both eyes twice daily. Despite this, an acute, severe pressure elevation was noted in the right eye 13 days post-operatively and in the left eye 14 days post-operatively. Over the following week, aqueocentesis was performed repeatedly (a total of three times) to relieve the intraocular hypertension, two episodes of which were facilitated using propofol for sedation (total dose each time was 0.5 mg/kg). During both instances, he did not develop any clinical signs suggestive of hyperkalemia, but his electrolytes were not rechecked immediately post-procedure.

**Table 4:** Chemistry panel submitted by CUVS 2 weeks following anesthesia.

Albumin (g/dL) (RI 23–40 g/dL)	20
ALT (U/L) (RI 10–125 U/L)	57
ALP (U/L) (RI 23–212 U/L)	113
Glucose (mmol/L) (RI 4.07–7.86 mmol/L)	17.3
Cholesterol (mmol/L) (RI 2.86–8.55 mmol/L)	12.7
Amylase (U/L) (RI 500–1,500 IU/L)	406
Phosphorus (mmol/L) (RI 0.8–2.19 mmol/L)	2.22
Calcium (mmol/L) (RI 1.97–2.99 mmol/L)	2.07

*RI, reference interval; ALP, alkaline phosphatase; ALT, alanine transaminase*.

The patient was then presented to Central Hospital For Veterinary Medicine 21-days post-operatively for bilateral endolaser cyclophotocoagulation to treat refractory glaucoma. Pre-operative point of care bloodwork^29^ was within normal limits (Table 5). A peripheral intravenous catheter was placed. He was premedicated with hydromorphone^30^ (0.1 mg/kg) and midazolam (0.2 mg/kg) IV. General anesthesia was induced with propofol (1.06 mg/kg) IV to allow endotracheal intubation. The patient was connected to a rebreathing anesthetic circuit with isoflurane as the inhalant anesthetic and he was mechanically ventilated throughout the procedure. Fluid therapy was provided with lactated Ringer's solution^31^ at 10 mL/kg/hr. Atracurium^32^ (0.2 mg/kg) was administered 30 min following induction and was reversed with neostigmine^33^ (0.02 mg/kg) IV 150-min following induction. Two additional bolus doses of propofol (0.6 mg/kg each) were administered IV intra-operatively at 60- and 90-min following induction to maintain adequate depth of anesthesia. The patient remained hemodynamically stable throughout the surgery but upon recovery, point of care bloodwork was checked which revealed hyperkalemia with relative hyponatremia (Table 5). Regular insulin (0.1 IU/kg), 50% dextrose^34^ (100 mg/kg) and 0.9% NaCl^35^ (0.4 mL/kg) were given IV. Eight hours later, the hyperkalemia resolved (Table 5). An abdominal ultrasound was repeated to investigate possible causes of recurrent hyperkalemia, and the findings were unchanged from previous study 21 days prior. A resting cortisol level was sent to an external laboratory^28^ at this time and was now low (8.28 nmol/L, reference interval 55.2–165.6 nmol/L); however, this result was suspected to be influenced by the fact that the patient had been treated with ophthalmic prednisone acetate drops. Hypoadrenocorticism was considered unlikely in this patient due to a previously reported normal cortisol level, normal ACTH stimulation test 3 weeks prior, and static ultrasonographic appearance of the adrenal glands reported on two separate occasions 3 weeks apart. A fasted triglyceride level was checked to rule out marked hypertriglyceridemia causing pseudohyperkalemia and was mildly elevated (1.96 mmol/L, reference interval 0.22–1.65 mmol/L). The patient recovered well and was discharged 6 days post-operatively.

**Table 5:** Point of care bloodwork (venous) at Central Hospital For Veterinary Medicine.

**Date/Time**	**12/26/2018** **15:00** **24-h prior to anesthesia**	**12/27/2018** **15:19** **Immediately following anesthesia**	**12/27/2018** **20:11** **5-h following anesthesia**	**12/28/2018** **00:07** **9-h following anesthesia**	**12/28/2018** **13:25** **21-h following anesthesia**
HCT (%) (RI 35–50%)	32	25	30	30	31
BUN (mmol/L) (RI 3.57–9.28 mmol/L)	5.7	4.64	5.36	5.7	5.7
Creatinine (umol/L) (RI 44.2–114 umol/L)	79.5	79.5	61.8	61.8	70.7
iCa (mmol/L) (RI 1.12–1.4 mmol/L)	1.15	1.25	1.26	1.27	1.23
Glucose (mmol/L) (RI 3.3–6.3 mmol/L)	5.7	20.5	28.9	16	22
Sodium (mmol/L) (RI 142–150 mmol/L)	145	136	136	144	141
Potassium (mmol/L) (RI 3.4–4.9 mmol/L)	4.5	8	6.2	4.5	4.8
Chloride (mmol/L) (RI 106–127 mmol/L)	118	113	114	114	115

*RI, reference interval; HCT, hematocrit; BUN, blood urea nitrogen; iCa, ionized calcium*.

Due to the development of repeated intra-operative hyperkalemia at two different facilities, propofol infusion syndrome was considered a potential cause for the acute hyperkalemia. Given this concern, when the patient underwent a third anesthetic episode for bilateral enucleation at CUVS 7 weeks later, alfaxalone^36^ (1 mg/kg) IV was used as an anesthetic induction agent instead of propofol. Methadone (0.2 mg/kg) and midazolam (0.2 mg/kg) were used as premedication. General anesthesia was maintained using isoflurane inhalant anesthetic but no neuromuscular blocking agents were used. Pre- and post-operative electrolyte levels remained within normal limits throughout this hospital stay. The patient's diabetes mellitus was better controlled during this visit: blood glucose was 11.5 mmol/L pre-operatively and 19.7 mmol/L post-operatively. The patient had an uneventful recovery and was discharged the following day. No further hyperkalemic episodes were suspected or documented.

Five months after his cataract surgery, the patient was diagnosed with a rapidly progressive C7–T1 myelopathy. At that time, considering the patient's multitude of health issues and concerns for his overall poor prognosis, his owners elected humane euthanasia.

## Discussion

This report describes the repeated development of marked hyperkalemia during general anesthesia for ophthalmologic surgery in a dog. There are many causes of intra-operative hyperkalemia, broadly divided into the following categories: altered potassium distribution (e.g., increased potassium release from cells or other transcellular shifts, including severe metabolic acidosis, thrombocytosis, hemolysis, rhabdomyolysis) (1, 5), reduced renal/urinary excretion (e.g., uroabdomen, administration of potassium-sparing diuretics, intravascular volume depletion, hypoaldosteronism/hypoadrenocorticism), malignant hyperthermia, an increased exogenous potassium load (e.g., drug-related, iatrogenic potassium chloride injection) or parasitic infestation (whipworms). There are also reports of veterinary species-specific problems such hyperkalemic periodic paralysis in Quarter Horses (6) [also reported in a dog (7)], episodic hyperkalemia in Greyhounds (8), and unexplained hyperkalemia in non-domestic felids (9). There was a recent case series documenting repeated intra-operative hyperkalemia in two Greyhounds where the inciting cause was not identified (2). Greyhounds reportedly have significantly lower than average basal aldosterone levels, which may have contributed to the development of hyperkalemia or to their inability to rapidly and/or effectively respond to increases in serum potassium when they occurred. These dogs also received medetomidine which could contribute to hyperkalemia due to the inhibitory effects of alpha-2 adrenergic receptor agonists on the production of insulin (2). Our patient was neither a Greyhound nor did he receive an alpha-2 adrenergic agonist, therefore the above hypothesis is unlikely to be applicable in this case.

The patient in this report had a prior history of mild pseudohyperkalemia secondary to thrombocytosis. However, this typically results in a mild increase in potassium levels (0.3–0.5 mmol/L above baseline) secondary to increased potassium release from activated platelets. In the absence of hemolytic serum and elevated bilirubin levels, intravascular hemolysis appeared unlikely. The patient did not receive any drugs that would impair potassium excretion and the risk of iatrogenic injection of potassium chloride was considered negligible—all fluid bags and medications administered were rechecked, and the fact the hyperkalemia recurred at a different veterinary facility made this unlikely. Rhabdomyolysis was considered unlikely given the lack of pigmenturia observed on both occasions and a normal creatinine kinase level obtained 3 days after the initial event. Acute kidney injury was ruled out by normal urea nitrogen and creatinine levels, and adrenal function testing did not support hypoadrenocorticism. Considering the above, metabolic causes were thought to be less likely and the focus shifted to drug-induced hyperkalemia.

Timolol was considered to be a potential contributing cause of hyperkalemia due to a single case report in human patients which described the development of severe hyperkalemia after administration of timolol (10). Timolol is a beta-antagonist that can impair potassium homeostasis by reduced sodium-potassium-ATPase activity, preventing potassium influx and sodium efflux into cells and leading to development of hyperkalemia. Given the scarce reports of beta-antagonist induced hyperkalemia in human patients, the authors believe timolol was unlikely to be the cause of the hyperkalemia. Other medications that the patient received on both occasions included midazolam, isoflurane inhalant, propofol, and non-depolarizing neuromuscular blocking agents. Extensive literature review revealed no case reports or known associations between midazolam and the development of hyperkalemia. Isoflurane-induced malignant hyperthermia (MH) leading to hyperkalemia has been reported in both human medicine and in a dog (11). MH is thought to be an autosomal dominant inherited disease caused by a mutation of *RYR*1 gene. The most common features of MH in dogs are hypercarbia, hyperthermia, and cardiac arrhythmias (11). Rhabdomyolysis is thought to be the cause of hyperkalemia in MH. Given the patient's multiple previous exposures to isoflurane, low-normal body temperature, and normal end-tidal carbon dioxide throughout both surgeries, malignant hyperthermia was considered highly unlikely.

Propofol infusion syndrome (PRIS) has been reported in human medicine (12, 13) and in one veterinary case report (3). This is a syndrome occurring in critically ill patients receiving propofol infusions, typically at high doses (>5 mg/kg/h) or prolonged infusion (>48 h), and is characterized by one of the following changes that are otherwise unexplained: metabolic acidosis, rhabdomyolysis, or ECG changes, with or without AKI, hyperkalemia, hyperlipidemia, cardiac failure, elevated liver enzymes, or raised serum lactate (12, 13). There are also human case reports documenting hyperkalemia after a single bolus of propofol at an average dose (14, 15). There have been a few recent case reports published describing development of peri-operative hyperkalemia in dogs and all of these patients received propofol (2–4). However, aside from the case reported by Mallard et al. (3), none of the dogs developed signs supportive of PRIS except for hyperkalemia and did not receive a continuous rate infusion. The patient in our study had received propofol at varying doses (0.5–4 mg/kg) on eight occasions previously and had no hemodynamic changes consistent with PRIS. However, it is still possible that the signs noted during these two events was related to early PRIS, or was a precursor for the development of PRIS, since a dose-dependent relationship has been suggested in human medicine (12).

Another recent report described development of marked hyperkalemia intra-operatively in a dog with poorly controlled diabetes mellitus undergoing anesthesia for phacoemulsification (4). This patient underwent the same procedure (elective phacoemulsification), received propofol as an induction agent, was maintained on inhalant isoflurane anesthesia, and received a non-depolarizing neuromuscular blocking agent (atracurium). An additional similarity between this dog and the patient in our study was that both animals were diabetic patients that did not have optimal glycemic control. However, the diabetic patient described in the previous report also received medetomidine, which may have contributed to the hyperkalemia noted. In that report, the patient's hyperkalemia was presumed to be caused by poorly controlled diabetes mellitus leading to a combination of insulin deficiency and hyperosmolality, resulting in hyperkalemia through fluid shifts from the intracellular to extracellular compartment (4). A normal fructosamine level was documented pre-operatively in our patient; however, moderate hyperglycemia was documented in the post-operative period following development of hyperkalemia. The patient had previously been exposed to propofol on multiple occasions (prior splenectomy, spinal surgery, endoscopy, and abdominal explore); however, these instances occurred before his diagnosis of diabetes mellitus (he had been diagnosed 7 months prior to his cataract surgery). There is evidence in animal models to suggest that propofol induces whole body insulin resistance and causes glycogen synthase kinase 3β-related mitochondrial dysfunction and apoptosis, and the link between insulin resistance and mitochondrial dysfunction has been well-described (16, 17). These studies have suggested that propofol-induced insulin resistance may contribute to the development of PRIS. The authors hypothesize that once this patient became a diabetic with altered carbohydrate metabolism, he may have been more susceptible to the development of mitochondrial dysfunction which could have mediated the development of early PRIS in this case on both occasions. This may explain why hyperkalemia or other signs suggestive of PRIS were not documented during previous exposures to propofol in this patient.

Neuromuscular blocking agents were also considered potential causes for our patient's hyperkalemia. There are well-documented reports of succinylcholine-induced hyperkalemia in human patients (18, 19) and one case report in an experimental study in dogs (20). Succinylcholine is a depolarizing neuromuscular blocking agent that differs from non-depolarizing agents in that it results in prolonged, irreversible binding at the postsynaptic acetylcholine (ACh) receptors. Succinylcholine has been reported to cause a transient, mild increase in potassium concentration up to 0.5 mmol/L above baseline (18). However, this effect can be exacerbated when the Ach receptors on skeletal muscle are upregulated or if denervation occurred such that the constituent subunits of the Ach receptor were altered (19). Both the patient in the aforementioned case report (4) and this patient received non-depolarizing neuromuscular agents, either cisatracurium or atracurium. There are no current human or veterinary reports of hyperkalemia developing after administration of either of these medications. However, the potential for non-depolarizing neuromuscular blocking agents to have contributed to intra-operative hyperkalemia cannot be entirely ruled out.

Given these considerations, the authors hypothesize that this patient's repeated, marked intra-operative hyperkalemia was most likely associated with propofol, although other contributing causes such as suboptimal glycemic control of diabetes mellitus and contributions from other medications cannot be ruled out completely. Hyperkalemia can be a life-threatening emergency and is an important differential in bradycardic patients under anesthesia that are not responsive to traditional therapies. Administration of calcium gluconate to increase the cardiac threshold potential and administration of insulin with dextrose, beta-2 agonists or sodium bicarbonate to encourage the intracellular shift of potassium are interventions that should be considered to reduce the risk of fatal arrhythmias and to decrease circulating potassium concentrations. Although a definitive underlying cause could not be identified in this patient, this case report adds to current veterinary literature by raising awareness of the potential for development of severe intra-operative hyperkalemia that could be related to various anesthetic medications. Close monitoring and prompt identification of intra-operative hyperkalemia is vital for rapid intervention to treat this life-threatening anesthetic complication.

## Data Availability Statement

The raw data supporting the conclusions of this article will be made available by the authors, without undue reservation, to any qualified researcher.

## Ethics Statement

The case report is a retrospective evaluation with no active interventional or research component, therefore ethical approval was not indicated. Client consent was not obtained as the data provided in the following case report does not contain any identifiable information.

## Author Contributions

CT and AB participated in the manuscript preparation. RW participated in critical revisions of the manuscript.

## Conflict of Interest

The authors declare that the research was conducted in the absence of any commercial or financial relationships that could be construed as a potential conflict of interest.

## Footnotes

^1^Zoasis Laboratory: Antech Diagnostics, Inc., Los Angeles, CA.

^2^NPH insulin: Lilly, Indianapolis, IN.

^3^Ketorolac Tromethamine 0.5% Ophthalmic Solution: Akorn Inc., Lake Forest, IL.

^4^Prednisolone Acetate 1% Ophthalmic Suspension USP: Alcon Vision LLC, Fort Worth, TX.

^5^GenTeal Tears: Alcon Vision LLC, Fort Worth, TX.

^6^Tropicamide 1% Ophthalmic Solution USP: Valeant Pharmaceuticals North America LLC, Bridgewater, NJ.

^7^Neomycin and Polymyxin B Sulfates and Gramicidin Ophthalmic Solution, USP: Valeant Pharmaceuticals North America LLC, Bridgewater, NJ.

^8^Phenylephrine Hydrochloride 2.5% Ophthalmic Solution, USP: Akorn Inc., Lake Forest, IL.

^9^Dorzolamide HCl 2%/Timolol Maleate 0.5% Ophthalmic: Valeant Pharmaceuticals North America LLC, Bridgewater, NJ.

^10^I.V. catheter: Terumo (Phillipines) Corporation, Laguna, Philippines.

^11^Methadone: Mylan Institutional LLC, Rockford, IL.

^12^Midazolam: Akorn Inc., Lake Forest, IL.

^13^Propofol: Zoetis, Kalamazoo, MI.

^14^Isoflurane: Henry Schein, Dublin OH.

^15^Cefazolin: West-Ward, Eatontown, NJ.

^16^LifeWindow: Digicare Biomedical, Boynton Beach, FL.

^17^Cisatrucurium: AbbVie Inc, North Chicago IL.

^18^Plasmalyte-A: Baxter Healthcare Corp, Deerfield, IL.

^19^Glycopyrrolate: West-Ward, Eatontown, NJ.

^20^Atropine: Vet One, Boise, ID.

^21^Naloxone: Akorn Inc., Lake Forest, IL.

^22^Flumazenil: West-Ward, Eatontown, NJ.

^23^RapidPoint 500: Siemens, Munich, Germany.

^24^10% Calcium gluconate: Fresenius Kabi, Lake Zurich, IL.

^25^Regular insulin: Lilly, Indianapolis, IN.

^26^Terbutaline: West-Ward, Eatontown, NJ.

^27^IDEXX VetLab Station: IDEXX Laboratories, Westbrook, ME.

^28^IDEXX Laboratories, Westbrook, ME.

^29^Hydromorphone: West-Ward, Eatontown, NJ.

^30^VetScan i-STAT: Abaxis Inc., Union City, CA.

^31^Lactated Ringer's Solution: Dechra, Overland Park, KS.

^32^Atracurium Besylate: Schaumburg, IL.

^33^50% dextrose: Vet One, Boise, ID.

^34^0.9% NaCl: Hospira, Lake Forest, IL.

^35^Alfaxalone: Jurox Inc., North Kansas City, MO.

^36^Neostigmine: Amphastar Pharmaceuticals Inc, Rancho Cucamonga, CA.
